# Immune Activation Markers in Peripartum Women in Botswana: Association with Feeding Strategy and Maternal Morbidity

**DOI:** 10.1371/journal.pone.0089928

**Published:** 2014-03-21

**Authors:** Elizabeth S. Russell, Terence Mohammed, Laura Smeaton, Baitshepi Jorowe, Iain J. MacLeod, Risa M. Hoffman, Judith S. Currier, Sikhulile Moyo, Max Essex, Shahin Lockman

**Affiliations:** 1 Harvard AIDS Initiative, Department of Immunology and Infectious Diseases, Harvard School of Public Health, Boston, Massachusetts, United States of America; 2 Botswana-Harvard AIDS Institute, Gaborone, Botswana; 3 Center for Biostatistics in AIDS Research, Department of Biostatistics, Harvard School of Public Health, Boston, Massachusetts, United States of America; 4 David Geffen School of Medicine, University of California Los Angeles, Los Angeles, California, United States of America; 5 Department of Medicine, Division of Infectious Diseases, Brigham and Women's Hospital, Boston, Massachusetts, United States of America; Emory University School of Medicine, United States of America

## Abstract

Hormone levels shift the immune state in HIV-uninfected pregnant and breastfeeding women away from Th_1_ responses and toward regulation to permit fetal tolerance. Limited data exist on inflammation during pregnancy or postpartum in HIV-infected women, though certain inflammatory markers are associated with adverse health outcomes among HIV-infected persons.

We measured hsCRP, D-dimer, IFN-γ, IL-6, IL-10 and TNF-α at 34 weeks gestation and six months postpartum in HIV-infected women from the Botswana Mashi PMTCT trial who were randomized to breastfeeding or formula-feeding. Differences in inflammatory markers between gestation and postpartum periods, and by randomized feeding method, were estimated using generalized estimating equations, adjusting for baseline plasma HIV-1 viral load, CD4 count, calendar time, and antiretroviral treatment status. Additionally, we studied the association between marker concentrations at six months postpartum and major adverse clinical events over the following 4.5 years, using case-cohort sampling and adjusted Cox proportional hazards models.

In 86 breastfeeding and 75 formula-feeding women, hsCRP and D-dimer decreased significantly between 34 weeks gestation and six months postpartum, while IFN-γ increased. There was no significant association between inflammatory marker change and randomized feeding method after adjusting for multiple comparisons and removing outliers. In univariate analysis, TNF-α, D-dimer, and IFN-γ concentrations at six months postpartum were significant predictors of subsequent clinical events, and TNF-α remained significant in multivariate analysis (HR = 4.16, p = 0.001).

In young HIV-infected women in Botswana inflammatory marker concentrations did not differ significantly between women who breast- vs. formula-fed. However, postpartum TNF-α level was predictive of subsequent adverse clinical event.

## Introduction

Pregnancy and breastfeeding are associated with modulation of the immune system toward immune regulation and reduced Th_1_ responses [1], [2]. Hormonal changes during pregnancy, which persist in part during breastfeeding, are one cause of this immune shift [3]; breastfeeding and the hormone prolactin have been shown to be associated with B-cell production and reduced inflammatory responses [4], [5]. Such data support a hypothesis that breastfeeding could, at least transiently, influence HIV-1 disease progression through regulation of the immune response. Some, but not all [6], [7], [8], studies suggest that breastfeeding is associated with more rapid CD4 count decline or adverse health outcomes among HIV-infected women [9]. In one trial conducted in Kenya among HIV-infected women without access to antiretroviral treatment (ART), women randomized to breastfeeding had a higher mortality at two-years postpartum compared with those randomized to formula-feeding (10.5% vs. 3.8%, respectively) [10]. Similarly, a trend toward more rapid progression to AIDS or death was observed at six months postpartum in women randomized to breastfeeding in the Botswana Mashi trial, possibly as a result of higher maternal inflammation as suggested by CRP level, despite similar micronutrient level [11]. The cause of this potentially higher rate of HIV disease progression and adverse clinical outcomes associated with breastfeeding among HIV-infected women is unknown, and to our knowledge there are no published data on the association between inflammation and breastfeeding in HIV-infected women.

Levels of certain inflammatory markers, such as C-reactive protein, IL-6, and D-dimer, have been independently associated with subsequent morbidity and mortality in HIV-1-infected individuals in some [12], [13], [14], [15], [16], [17], [18] but not all [19] studies. However, many of these study populations were predominantly comprised of older men, and few studies included data from women of childbearing age or postpartum women [13]. The potential for a predictive role of inflammatory markers in HIV disease progression has not been explored in HIV-infected women from resource-limited settings, where other co-morbidities (including co-infections) may render these markers less informative.

Using stored samples from participants in a previously-completed randomized trial in Botswana that included feeding and antiretroviral interventions to prevent mother-to-child HIV transmission (the Mashi study) [20], we investigated two distinct aims: 1) the relationship between markers of inflammation between the third trimester and six months postpartum overall and by randomized feeding strategy (breastfeeding vs. formula-feeding); and 2) whether concentrations of inflammatory markers in otherwise healthy HIV-infected women at six months postpartum correlated with morbidity and mortality over a subsequent 4.5-year follow-up period.

## Methods

### Participants

The study was approved by the IRBs of both the Harvard School of Public Health and the Botswana Ministry of Health. Women provided written informed consent for study participation and storage/use of samples/data. We used stored samples and data from the completed Mashi mother-to-child HIV transmission (MTCT) prevention trial in Botswana (ClinicalTrials.gov registration number NCT00197587). The Mashi trial randomized 1200 HIV-infected women to six months of breastfeeding plus six months of infant zidovudine prophylaxis, or to formula-feeding plus infant zidovudine for one month, as previously described [20]. Women in the breastfeeding arm were asked to exclusively breastfeed for five months then initiate weaning in order to fully wean to formula by six months postpartum. Women were enrolled between 33 and 35 weeks' gestation during 2001 to 2003, and were followed for up to five years postpartum. HIV RNA plasma viral load and CD4 cell count were tested at enrollment (∼34 weeks gestation), delivery, and then approximately every six months postpartum for at least two years (plasma was stored at these time points). All women received zidovudine from enrollment through delivery; in 2002 (19 months into enrollment), three-drug ART became available and was offered to all HIV-infected infants and to all women with a CD4 count <200 cells/µl or history of an AIDS-defining illness. Women provided written informed consent for study participation and storage/use of samples/data.

### Study design

**Figure 1:**
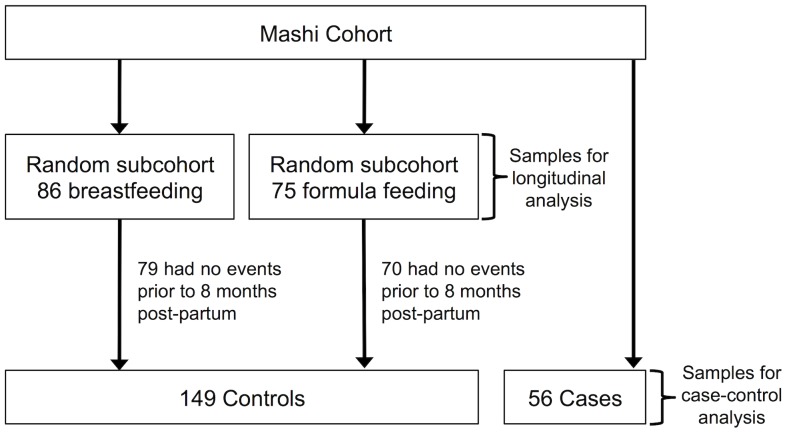
Selection of subcohorts. For Aim 1, we selected at random 86 women from the breastfeeding and 75 women from the formula-feeding arms of the Mashi Study. For Aim 2, a case was defined as a woman who had a six-month postpartum sample available, and experienced a major adverse clinical event after, but not before, eight months postpartum. Women randomly selected for Aim 1 who did not experience any adverse clinical events comprised the controls for Aim 2.

The first part of this study was to characterize changes in inflammatory markers over time in each feeding arm - from 34 weeks gestation to six months postpartum - and to compare levels of inflammatory markers between randomized feeding arms at both time points. The estimated sample size needed to provide 80% power to detect a difference of one standard deviation in most marker levels at alpha = .05 was 67 per randomized feeding arm. From the Mashi cohort we selected a random sample of 86 women in the breastfeeding arm and 75 women in the formula feeding arm among those with plasma samples available at both enrollment and six months postpartum (PP) (Fig. 1).

The second part of this study was to analyze whether inflammatory marker concentration at six months postpartum predicted subsequent major adverse clinical outcomes, using a case-cohort study design [21]. An adverse clinical event was defined as death, an AIDS-defining illness, or a diagnosis of grade 3 or 4 (severe or life-threatening) illness (Table 1). Grade 3 or 4 conditions that were assumed to have no relationship to inflammation and/or HIV disease were excluded (e.g. repeat pregnancy, injury, migraine headache). A case was defined as a woman who had a six-month postpartum sample available for testing and experienced a major adverse clinical event after eight months postpartum but no previous major adverse clinical event documented by the study team prior to this time. Women in the cohort were those selected for the first study aim who also did not experience any adverse clinical events prior to eight-months postpartum in order to reduce the likelihood that biomarker concentrations at six months were influenced by concurrent illness (n = 149, Fig. 1; five formula-feeding and seven breastfeeding women who experienced illness prior to eight months were excluded from this case-cohort sample). With 10% of participants experiencing a qualifying adverse clinical outcome in the total cohort, a subcohort size of 12% (n = 144) would have 80% power to detect a hazard ratio (HR) of 1.4 [22].

**Table 1:** Case diagnoses.

Grade 3/4		AIDS-Defining	Death
Peripheral Neuropathy (n = 2)^a^	Persistent HPV- LGSIL	TB-related diagnosis (n = 20)^e^	Eclampsia
Abscess	CIN 3	Esophageal Candidiasis	Renal Failure
Neutropenia	Cervical Carcinoma in situ	Cervical Cancer	Hepatic Encephalopathy
Dysfunctional Uterine Bleeding	Cellulitis (n = 2)	Squamous Cell Carcinoma	Anemia
Generalized Blistering Rash	Cervicitis (n = 3)^d^		Chronic Gastroenteritis
Acute Otitis Media^b^			Meningitis
Herpes Zoster (n = 2)			Bacterial Meningitis
Anal Fissure			Unknown
Kaposi Sarcoma^c^			

a One participant additional diagnosis of gangrene, the other rectal divercation one month later.

b Previous diagnosis with ovarian cyst.

c Three months later sepsis.

d One simultaneously diagnosed with gastroenteritis, another later diagnosed with cryptococcal meningitis.

e Two participants with TB-related diagnoses had multiple diagnoses at the same visit (lymphadenitis, pleural effusion); one participant had an additional diagnosis of cervicitis 15 months later and meningococcal meningitis 32 months later.

### Laboratory Analysis

Blood plasma samples were tested for interleukin-6 (IL-6), tumor necrosis factor alpha (TNF-α), and interleukin-10 (IL-10) by ultrasensitive ELISA (Invitrogen Life Sciences, Carlsbad, CA); interferon-gamma (IFN-γ) by standard ELISA (Invitrogen Life Sciences, Carlsbad, CA); D-dimer by Cobas Integra 400 System (Tina-quant D-Dimer Gen.2); and high sensitivity C-reactive protein (hsCRP) by protein-latex assay (Roche, Basel, Switzerland). All ELISA and Cobas reagents were from the same lot for all tests. Samples were not divided by analysis subgroup during testing to reduce potential confounding due to variability between assay runs.

### Statistical Analysis

Statistical calculations were performed using STATA (version 8.2 for Macintosh, College Station, TX), and SAS (version 9.3, Cary, NC). For Aim 1 we used generalized estimating equations (GEE) to compare inflammatory marker concentrations over time and whether feeding arm was significantly associated with changes over time, using a binomial link for dichotomous and an identity link for continuous markers (each marker in a separate equation). To account for differences between feeding arms at enrollment, and previously published associations between plasma viral load/CD4 T cell count and inflammation, all models were adjusted for CD4 T cell count, HIV-1 plasma viral load, calendar time, and for ART initiation between enrollment and six months postpartum. As a high proportion of samples had concentrations below the level of detection (62% for IFN-γ, 69% for IL-10, and 41% for TNF-α), these were modeled as detected/undetected dichotomous observations. Breastfeeding and undetected inflammatory marker, where applicable, were the referents in all models. Although feeding strategy was randomized, mean enrollment values of hsCRP and TNF-α were significantly different at baseline between the strata. Sensitivity analyses including these variables were performed for all models. The main model outcomes were time (34 weeks gestation or six months postpartum), group (feeding strategy arm), and group*time (interaction) covariates. For IL-6, a small group of influential observations (n = 11, Pearson residuals <−4) had values near the lower limit of detection of the assay, and removing these outliers influenced the statistical significance of the time covariate (from a p-value of .037 to .23) and group*time interaction (from a p-value of <.001 to .08). When compared to the rest of the subcohort, the outliers had no unique characteristics: no common test date, assay batch, or clinical characteristics of the participants. The significant influence of this small number of observations led us to remove them from our analyses, but kept them in the calculation of means for completeness in our reporting.

Aim 2 (association of inflammatory markers with subsequent morbidity/mortality) was investigated using Cox Proportional Hazards models with appropriate weighting for our case-cohort design [23]. Univariate and multivariate models were fit for each proinflammatory marker. The adjusted models included the following covariates: maternal age at enrollment, six month postpartum HIV RNA viral load, six month postpartum CD4 T cell count, randomized feeding arm, and delivery calendar time represented by 12 three-month periods (calendar time of delivery adjusts for potential changes in public health practice).

## Results

### Participant Characteristics

We selected at random 86 women from the breastfeeding and 75 women from the formula-feeding arms of the Mashi Study. Women in this subcohort had slightly a higher median CD4 count (393 vs. 355 cells/µl, respectively, p = .04) and lower median HIV-1 RNA (4.2 vs. 4.4 log_10_ RNA copies/ml respectively, p = .05) at enrollment at 34 weeks gestation, compared with all women participating in the Mashi trial, but were similar in age and sites of enrollment (data not shown). Between breastfeeding and formula-feeding arms within the subcohort, there was no difference at enrollment in median CD4 count (p = .18), or in median viral load (p = .13) (median concentrations in Table 2, p-values between arms not shown). Similar clinical characteristics were seen in both arms at six months postpartum (Table 2).

**Table 2:** Patient characteristics at 34 weeks gestation and six months post-partum.

		Breastfeeding				Formula Feeding		
	n	34 Weeks Gestation	6 months post-partum	*p* value^c^	n	34 Weeks Gestation	6 months post-partum	*p* value^c^
Viral load (log_10_ RNA copies/ml)^a^	83	4.25 (4.1, 4.4)	4.20 (4.0, 4.4)	.79	71	4.1 (3.9, 4.3)	4.0 (3.8, 4.2)	.71
CD4 count (cells/µl)^a^	81	448 (393, 503)	440 (386, 494)	.84	67	398 (354, 442)	472 (416, 527)	.01
CRP (µg/mL)^a^	78	4.24 (1.8, 9.8)	3.02 (1.1, 4.8)	.009	68	3.21 (1.8, 5.5)	1.50 (0.59, 4.0)	.002
IL-6 (pg/mL)^a^	84	0.50 (0.28, 0.91)	0.54 (0.12, 1.2)	.58	75	0.41 (0.17, 0.99)	0.87 (0.48, 1.6)	<.001
D-dimer (pg/mL)^a^	73	1.4 (0.94, 2.4)	0.40 (0.29, 0.57)	<.001	66	1.32 (0.93, 1.8)	0.52 (0.31, 0.67)	<.001
TNF-α (%)^b^	81	68 (57, 78)	53 (42, 64)	.11	73	41 (29, 53)	55 (43, 66)	.03
IFN-γ (%)^b^	84	21 (13, 31)	46 (35, 58)	<.001	74	33 (23, 45)	62 (50, 73)	<.001
IL-10 (%)^b^	70	27 (18, 37)	24 (15, 36)	1.0	71	25 (16, 37)	37 (25, 49)	.09

a Median value (interquartile range).

b Median % of samples with detected concentration of marker (95% CI).

c *P*-values obtained from Wilcoxon signed-rank test between timepoints.

Between the formula and breastfeeding arms at enrollment, differences were found in the concentrations of TNF-α, 68% vs. 41% with detected concentrations in the formula and breastfeeding arms, respectively (p = .01), and hsCRP (4.24 vs. 3.21 ng/ml, p = .001). Only hsCRP was significant after adjusting for multiple comparisons (significant p<0.008). Sensitivity analyses including TNF-α and hsCRP as covariates did not alter the interpretation of the results described below. There were no differences in D-dimer, IFN-γ, IL-6 and IL-10 plasma concentration at enrollment between the two arms (data not shown).

**Figure 2:**
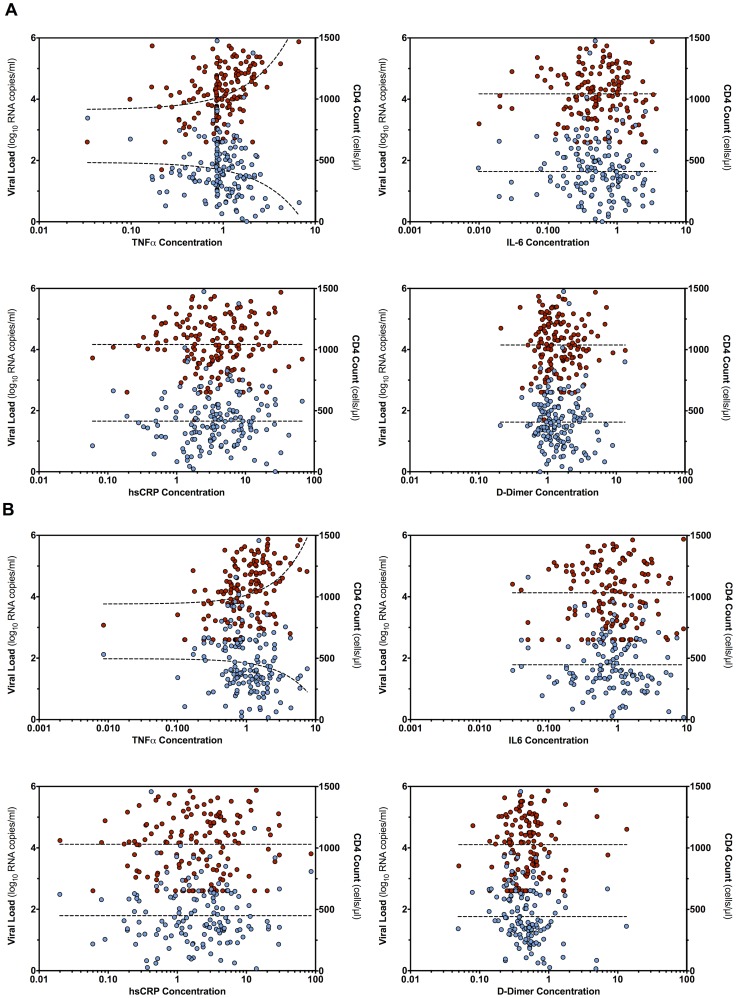
Association of viral load and CD4 count with cytokine expression. Viral load (red) is shown on the left y axis, and CD4 count (blue) on the right y axis. (a) association with cytokine expression at enrollment; (b) association with cytokine expression at six months postpartum.

TNF-α was significantly correlated with viral load at enrolment (r = .422, p<.001) (Fig. 2A) and six months postpartum (r = .412, p<.001) (Fig. 2B) as was CD4 count at both time points (r = −.170, p = .036 and r = −.231, p = .006, respectively); no other marker concentrations were associated with either viral load or CD4 T cell count.

### Markers of inflammation vary over time but not by infant feeding strategy

**Figure 3:**
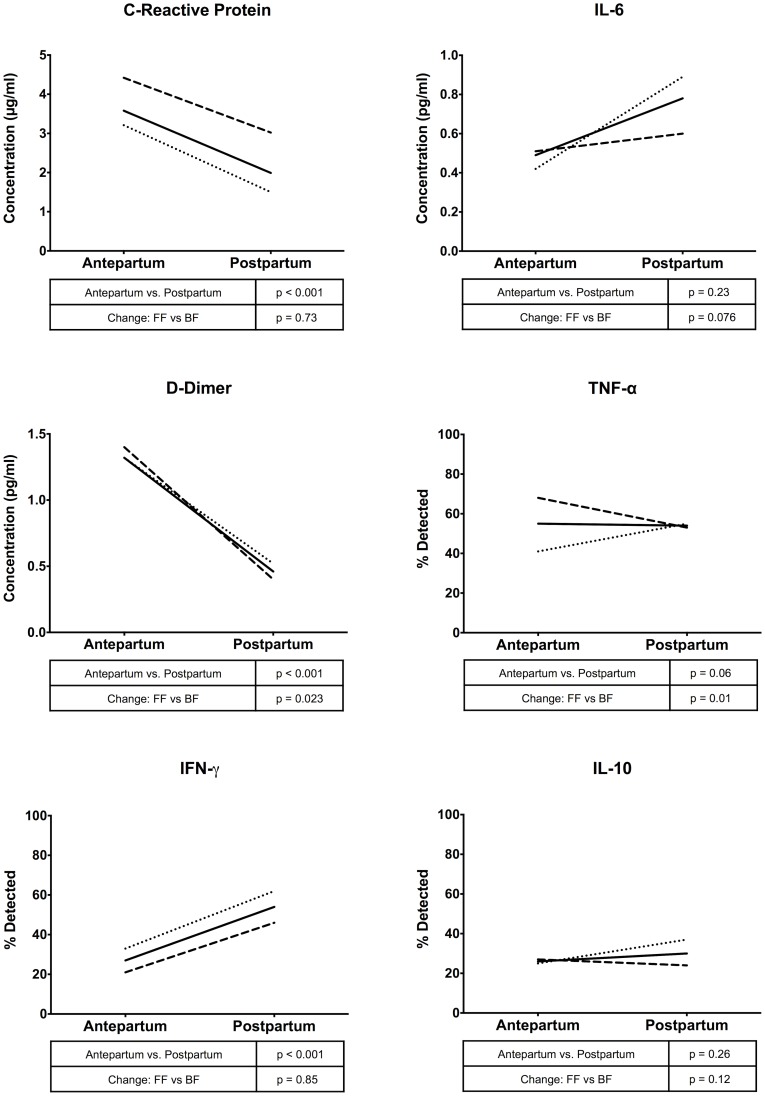
Changes in inflammatory marker concentrations between the third trimester and six months postpartum. The effect of time between enrolment (antepartum) and six months postpartum on inflammatory marker levels – either as absolute concentrations or percentage of women with detectable marker expression – was tested using generalized estimating equations (GEEs). Time and time*group interaction terms are reported, adjusted for CD4 and viral load at enrolment, and whether HAART was initiated between sampling times.

Changes in inflammatory marker concentrations from 34 weeks gestation to six months postpartum were similar in the breastfeeding and formula-feeding women after accounting for multiple comparisons (time*group interaction term p>.008 for all), demonstrating no effect of randomized infant feeding strategy on changes in biomarkers over time (Figure 3).

Concentrations of three out of the six markers of inflammation and coagulation tested were significantly different between 34 weeks gestation and six months postpartum overall: decreases were seen over time for hsCRP in the breastfeeding (4.24 vs. 3.02 ng/ml, nearly significant p = .009) and formula-feeding arms (3.21 vs. 1.50 ng/ml (p = .002) and also for D-dimer in the breastfeeding (1.40 vs. 0.40 ng/ml (p<.001) and formula-feeding arms (1.32 vs. 0.52 ng/ml, p<.001) (Table 2). IFN-γ increased in the breastfeeding (21 vs. 46 with detected concentrations (p<.001) and formula-feeding arms (33 vs. 62 with detected concentrations (p<.001, Table 2). Concentrations of the other three biomarkers, IL-6, IL-10, and TNF-α, after removing outliers and controlling for multiple comparisons, did not significantly change between the gestation and postpartum periods.

### TNF-α and IFN-γ concentrations at six months postpartum are associated with subsequent adverse clinical outcomes

In univariate models the hazard of experiencing an adverse clinical event increased significantly with a 1 pg/ml higher concentration of D-dimer (p = .007) and with detection of TNF-α at six months (p<.001) (Table 3). The hazard of a subsequent clinical event decreased with detection of IFN-γ at six months postpartum (p = .009) (Table 3). There was no significant association between the concentration of hsCRP, IL-10 or IL-6 and a subsequent clinical event in univariate analyses. For multivariate models, covariates known to be associated with both biomarker concentrations and clinical outcome were included: maternal enrollment plasma HIV-1 RNA load and CD4 T cell count, randomized feeding strategy, age, calendar time, and ART initiation during follow-up. Results of the multivariate model were similar, although higher D-dimer (p = .07) and lower IFN-γ (p = .01) were no longer statistically significantly associated with clinical outcome (with a significant p-value of 0.008 after considering multiple comparisons). TNF-α remained associated with adverse clinical events after considering multiple comparisons (p = .001) (Table 3). The multivariate model was a better fit than the univariate for all markers (by likelihood ratio test).

**Table 3:** Case-Control Study for Association of Inflammatory Marker Concentration and Major Adverse Clinical Events.

	Mean (IQR) Inflammatory Marker Concentration					Proportional Hazard Ratio for Major Adverse Clinical Event^a^					
	n	Case	n	Control	*p* value^b^	Univariate	95% CI	*p* value^b^	Multivariate^c^	95% CI	*p* value^b^
Viral load (log_10_ RNA copies/ml)	66	4.50 (3.4, 5.0)	129	4.18 (3.4, 4.8)	.25	-	-	-	-	-	-
CD4 count (cells/µl)	59	343 (265, 502)	123	406 (330, 612)	.05	-	-	-	-	-	-
hsCRP	64	2.16 (0.79, 5.6)	122	1.99 (0.80, 4.6)	.46	1.13	0.89–1.40	0.31	1.04	0.79–1.40	.76
IL-6 (pg/mL)	68	0.49 (0.12, 0.93)	132	0.76 (0.28, 1.3)	.03	0.91	0.81–1.00	0.11	0.93	0.79–1.10	.32
D-dimer (pg/mL)	62	0.49 (0.30, 1.03)	116	0.44 (0.30, 0.61)	.07	2.07	1.20–3.50	0.007	1.82	0.94–3.50	.07
TNFa (%)	66	80 (69, 89)	129	51 (42, 60)	<.001	3.60	1.80–7.10	<0.001	4.16	1.70–9.90	.001
IFN-g (%)	68	34 (23, 46)	132	55 (46, 63)	.005	0.45	0.25–0.82	0.009	0.37	0.17–0.82	.01
IL-10 (%)	62	48 (35, 61)	122	27 (19, 36)	.004	2.09	1.10–3.80	0.02	2.26	1.00–4.90	.04

a The model fit was a proportional hazards Cox model with appropriate weighting for the case-cohort design.

b *P*-values obtained from Wilcoxon signed rank (continuous) or Chi-Square (dichotomous) test.

c Adjusted for maternal age, calendar time of delivery, feeding strategy, HIV RNA load and CD4 count at 6 months post-partum.

## Discussion

Previously published studies have reported increased mortality or HIV disease progression among HIV-infected women randomized to breastfeeding [10], [13], with unknown underlying mechanisms to explain these findings. We present the first data examining the relationship of multiple inflammatory and coagulation biomarkers with excess mortality/AIDS associated with breastfeeding. We did not find a robust association between inflammatory and coagulation biomarkers and breastfeeding, which suggests that either the power in this study was not sufficient to detect an association, the availability of ART in this study may have influenced the results, or inflammation as measured by these six biomarkers may not explain these previous findings.

There are few published data describing inflammatory biomarkers in peripartum HIV-infected women, and those available are from US-based cohorts and each report on few markers and different timing of sample collection, providing little generalizability. Two studies of HIV-negative women found inflammatory markers increased from the first to the third trimester, with levels in one study returning to the first-trimester baseline by six months postpartum [24]. The hypothesis that inflammation would be further increased in the peri- and postpartum periods for HIV-positive women is supported indirectly by some reports that CD4 T cell counts declined during pregnancy for women on triple antiretroviral therapy [25] and HIV RNA increases from delivery through the first 80 weeks postpartum for women not on therapy or on mono- or combination therapy [26]. Viral load has been linked to increased inflammation [27], [28]. Direct evidence exists in one study that found higher concentrations of β2-microglubulin (but not TNF-α) in the third trimester of pregnancy and first weeks postpartum among HIV-infected women compared with HIV-uninfected controls [28], and the difference was identified up to six months postpartum, in a separate study [29]. These studies suggest that HIV-infected women experience changes in inflammatory marker concentrations during pregnancy beyond those seen in HIV-negative women with uncomplicated pregnancies, and these changes persist for months beyond delivery. We found a decline in hsCRP and D-dimer between the third trimester and six months postpartum, but an increase in IFN-γ. Given our finding that IFN-γ concentration was also associated with adverse clinical outcomes, these data would suggest a mechanism for this relationship.

In contrast to other study results [13], [15], [18], we did not find a significant association between hsCRP and subsequent adverse clinical events. One possible explanation is that other studies have examined populations that were exclusively or largely comprised of men, and these populations were also significantly older and individuals had more non-AIDS co-morbidities than Mashi participants. The Mashi study overall recorded few cardiovascular events as expected in this population of young women, and no cardiovascular events in this study population. More data exist for the association of standard, non high-sensitivity CRP and adverse outcomes in studies of populations similar to Mashi, and CRP is known to be associated with underlying infectious and inflammatory conditions [13], [30]. However, the predictive role of hsCRP is unclear. To minimize confounding from concurrent infections in our study, we excluded women with illness prior to or within two months after the time of hsCRP testing. While we may have unintentionally biased our selection toward a healthier population, it likely gives a cleaner analysis of the association of hsCRP elevation and subsequent adverse clinical events.

Prior studies have suggested that higher IL-10 and TNF-α concentrations were associated with HIV-1 disease progression in adult HIV-infection, as well as lower IFN-γ with lower viral load set point [31], [32]. We found similar trends between these cytokines and subsequent morbidity and mortality, independent of CD4 T cell count and viral load.

There are several limitations to this study. As samples were only collected and analyzed in the third trimester and at six months postpartum, we do not know the pre-pregnancy baseline or how marker concentrations changed during the first two trimesters of pregnancy or at the time of delivery. It is possible that an analysis of pre-pregnancy concentrations would yield different results. Antiretroviral treatment was also not assigned as part of the study, but given according to government guidelines. Our attempts to control for this in the analysis may leave residual confounding. Additionally, we looked at serum concentrations of six inflammatory marker proteins. An analysis of mRNA expression as cellular markers of immune activation [33], or different soluble markers such as markers of monocyte activation may have more accurately reflected chronic inflammation and immune activation in this population [34]. The markers we chose reflected previously published studies and allowed us to compare our results to those findings.

In summary, our data provide support for the need for further investigation of the role of inflammation in clinical outcomes among HIV-infected women peripartum (including the role of T cell activation and monocyte activation), and of strategies to reduce inflammation in the postpartum period. The randomized study, Promoting Maternal-Infant Survival Everywhere (PROMISE), is currently enrolling participants and aims to answer the question of whether there is a benefit to women with CD4 cell counts above current thresholds for treatment in continuing ART initiated for PMTCT [35]. If pregnancy-related immune modulation leads to an extended increase in inflammation postpartum (with associated adverse clinical outcomes), this would add evidence to support women remaining on ART after pregnancy, regardless of CD4 count, for their own health [36], [37].
